# Mediastinal cysts with Mullerian differentiation

**DOI:** 10.1002/rcr2.324

**Published:** 2018-05-03

**Authors:** Hiroyuki Miura, Jun Miura, Hiroshi Hirano

**Affiliations:** ^1^ Department of Thoracic Surgery Akiru Municipal Medical Center Tokyo Japan; ^2^ Department of Surgery Kyorin University School of Medicine Tokyo Japan; ^3^ Department of Pathology Hachioji Medical Center of Tokyo Medical University Tokyo Japan

**Keywords:** Mediastinal tumour, Mullerian cyst, oestrogen receptor, progesterone receptor

## Abstract

Mediastinal cysts with Mullerian differentiation are characterized by oestrogen receptor and progesterone receptor positive cell linings.

We treated four patients with mediastinal cysts with Mullerian differentiation. All patients were female, from 46 to 52 years old, and had BMIs greater than 26 kg/m^2^. All but one patient with cough were asymptomatic. The tumours were located in the paravertebral areas around the Th‐1–7 vertebrae. There was no left/right difference. All of the preoperative diagnoses were bronchial cysts. Three of the four patients had a history of uterine myoma. Furthermore, antiestrogen therapy was administered to one patient. All of our patients were alive without recurrence for three to eight years after surgery. As long as ectopic epithelia exist, there is a possibility of malignant conversion. Therefore, complete resection of the tumour is necessary.

## Introduction

“Mediastinal cysts with Mullerian differentiation” was a new category proposed by Hattori in 2005 1. This tumour is characterized by oestrogen receptor and progesterone receptor positive cells in the lining 2. We treated four patients with Mullerian cysts from 2008 to 2014. This is 4.3% of 93 patients with mediastinal tumours subjected to surgeries in the same period, and 10.3% of 39 patients with mediastinal cysts subjected to surgeries in the same period. Here, we report on the characteristics of the tumours of these four patients, as well as a summary of the reported cases.

## Case Series

### Case 1

A 50‐year‐old female patient presented to our hospital for cough during follow up after breast cancer surgery. The patient had received five years of hormonal therapy after undergoing a left mastectomy. The patient had a history of uterine myoma. A cystic tumour that was approximately 19 × 18 mm in size was observed in the left side of the posterior mediastinum, around the Th6–7 vertebrae. The patient’s body mass index (BMI) was 27.4 kg/m^2^.

### Case 2

A 52‐year‐old female patient presented with an abnormal shadow on a chest X‐ray during an annual check‐up. The patient did not have any notable medical history. A cystic tumour that was approximately 52 × 42 × 32 mm in size was observed in the right side of the posterior mediastinum, around the Th3–4 vertebrae. The patient’s BMI was 26.8 kg/m^2^.

### Case 3

A 46‐year‐old female patient was referred to our hospital due to an abnormal shadow on a chest X‐ray during an annual check‐up. The patient had a history of uterine myoma. A cystic tumour that was approximately 41 × 30 × 23 mm in size was observed in the right side of the posterior mediastinum, around the Th4–5 vertebrae. The patient’s BMI was 26.7 kg/m^2^.

### Case 4

A 52‐year‐old female patient presented to our hospital due to an abnormal shadow on a chest X‐ray during an annual check‐up. The patient had a history of uterine myoma. A cystic tumour that was approximately 28 × 18 × 30 mm in size was observed in the left side of the posterior mediastinum, around the Th1–2 vertebrae. The patient’s BMI was 36.2 kg/m^2^.

**Figure 1:**
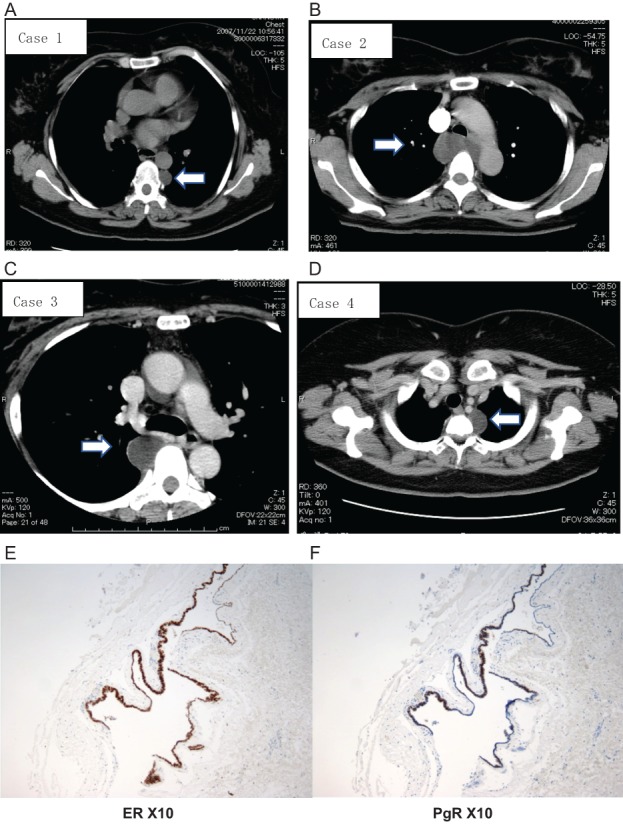
(A–D) Computed tomography scan of the chest showing each paravertebral tumour (arrow). (E, F) Immunohistochemical staining of case 4 (ER X10, PgR X10). The epithelial cells lining the inner side of the tumour were positive for ER and PgR.

All four patients were diagnosed as having a bronchial cyst, and the tumours were removed using video‐assisted thoracoscopic surgery. Pathological findings in these four patients were similar. The tumours were unilocular with thin‐wall cysts. Monotonous columnar cells without atypia lined the inner side of the cystic tumours. Cartilage cells, gland cells, and goblet cells were not observed. The cells in the lining were positive for oestrogen receptor (ER) and progesterone receptor (PgR) (Fig. 1) and negative for thyroid transcription factor 1 (TTF‐1). Malignant findings were not observed.

## Discussion

A total of 22 patients with Mullerian cysts were reported, including the four aforementioned patients 2, 3, 4, 5, 6, 7, 8 (Table 1). However, the cells in the lining of two of these patients were negative for ER and PgR (indicated by * in Table 1). Therefore, there were no signs typical of Mullerian differentiation in these two patients. Excluding these two patients, the 20 reported patients were all female, aged 18–56 years (mean 47.6 years). All but one 18‐year old patient were in there 40s or 50s. Half of the patients were asymptomatic. The chief complaints were cough or chest pain. The tumours were located in the left side in 11 and the right side in nine patients. There was no left/right difference. The tumours were in the paravertebral area, around the Th1–8 vertebrae. Tumour sizes ranged from 13 to 52 mm.

**Table 1:** Characteristics of Mullerian cyst in literature

	Sex	Age	Symptoms	Location		Size (mm)
Hattori	F	52	Persistent cough	R	Th6	25
	F	18	Asymptomatic	R	5	20
	F	49	Cough	L	4	20
Thomas‐de‐ Montpreville	F	40	Chest pain	L	4	15
	F	46	Cough	L	4	33
	F	47	Cough	R	4/5	50
	F	48	Asymptomatic	L	5	30
	F	50	Chest pain	R	3/4	32
	F	51	Asymptomatic	L	3/4	30
	F	56	Asymptomatic	L	8	13
	F	58	Cough	Pre	5	45*
	F	59	Chest pain	R	2–4	25*
Businger	F	54	Asymptomatic	L	4–6	45
Batt	F	41	Chest pain	L	6	21
Kobayashi	F	53	Asymptomatic	R	5	20
Liao	F	48	Chest tightness	R	6?	51
Simmons	F	52	Shortness of breath	R	6?	41
	F	47	Asymptomatic	L	?	50
Present cases	F	50	Cough	L	6/7	19
	F	52	Asymptomatic	R	3/4	52
	F	46	Asymptomatic	R	4/5	41
	F	52	Asymptomatic	L	1/2	30

* , patients negative for oestrogen receptor (ER) and progesterone receptor (PgR).

The proposed aetiology of the tumour included theories such as misplaced mesothelium with Mullerian characteristics 1 or misplaced endosalpingeal epithelium 5. The migration of the immature Mullerian duct into the mediastinum during the foetal period is more feasible than the differentiation of tumour cells into the Mullerian duct. All of our patients were obese and had BMIs greater than 25 kg/m^2^. The oestrogen level of the obese female is high due to aromatase existing in fatty tissue. Businger et al. 4 reported on a patient with Mullerian cysts under hormone replacement therapy with oestrogen and gestagen. Liao et al.^.^ 7 reported on tumours with high oestrogen levels. It is apparent that female hormones are involved in tumour growth. Three of the four patients had a history of uterine myoma. Furthermore, antiestrogen therapy was administered to one patient. The gynaecological disorder may be involved in the tumour development.

Pathologically, the tumours had thin walls with cuboidal or columnar cells in the inner side of the lining. These cells were positive for ER and PgR and negative for TTF‐1, indicative of Mullerian duct origin.

We performed surgeries on 93 patients with mediastinal tumours during these five years. There were 24 patients with thymomas, 23 with thymic cysts, seven with bronchial cysts, six with thymic cancers, five with pericardial cysts, four with Mullerian cysts, and the remaining patients had other types of tumours. The incidence of Mullerian cysts among those with mediastinal tumours was 4.3% and was 10.3% among those with mediastinal cysts at our institute. Although the rate of occurrence of mediastinal tumours is rare, careful examination including immunohistochemical tests may help to differentiate Mullerian cysts from mediastinal cysts. Many patients with Mullerian cysts may be misdiagnosed as having bronchial cysts. All of the preoperative diagnoses of Mullerian cysts were bronchial cysts. Bronchial cysts located at the paravertebral area are not rare and are not always associating with bronchus. Thomas‐de‐Montpreville and Dulmet reported 66 bronchial cysts 3. Among them, six tumours were located at the paravertebral area. They also reported nine Mullerian cysts that were all diagnosed as bronchogenic cyst preoperatively. It is difficult to distinguish Mullerian cysts from bronchial cysts from the shape and the site of occurrence. The paravertebral cysts in a middle‐aged female patient should be examined while considering the possibility of Mullerian cysts. As long as ectopic epithelia exist, there is a possibility of malignant conversion like the other migrated tumour. For example, the risk of testicular cancer is high in the patient with cryptorchidism 9, and thyroid cancer is more common in the mediastinal goiter 10. Therefore, complete resection of the tumour is necessary. The long‐term prognosis of Mullerian cysts is unclear. All of our patients were alive without recurrence from three to eight years after surgery. In order to clarify the characteristics of Mullerian cyst such as recurrence, accumulation, and follow‐up observation of the tumours is required.

## Disclosure Statement

Appropriate written informed consent was obtained for publication of this case series and accompanying images.
